# Mechanisms coupling sodium and magnesium reabsorption in the distal convoluted tubule of the kidney

**DOI:** 10.1111/apha.13528

**Published:** 2020-07-26

**Authors:** Gijs A. C. Franken, Anastasia Adella, René J. M. Bindels, Jeroen H. F. de Baaij

**Affiliations:** ^1^ Department of Physiology Radboud Institute for Molecular Life Sciences Radboud University Medical Center Nijmegen the Netherlands

**Keywords:** distal convoluted tubule, hypomagnesaemia, ion transport, kidney, magnesium, sodium

## Abstract

Hypomagnesaemia is a common feature of renal Na^+^ wasting disorders such as Gitelman and EAST/SeSAME syndrome. These genetic defects specifically affect Na^+^ reabsorption in the distal convoluted tubule, where Mg^2+^ reabsorption is tightly regulated. Apical uptake via TRPM6 Mg^2+^ channels and basolateral Mg^2+^ extrusion via a putative Na^+^‐Mg^2+^ exchanger determines Mg^2+^ reabsorption in the distal convoluted tubule. However, the mechanisms that explain the high incidence of hypomagnesaemia in patients with Na^+^ wasting disorders of the distal convoluted tubule are largely unknown. In this review, we describe three potential mechanisms by which Mg^2+^ reabsorption in the distal convoluted tubule is linked to Na^+^ reabsorption. First, decreased activity of the thiazide‐sensitive Na^+^/Cl^−^ cotransporter (NCC) results in shortening of the segment, reducing the Mg^2+^ reabsorption capacity. Second, the activity of TRPM6 and NCC are determined by common regulatory pathways. Secondary effects of NCC dysregulation such as hormonal imbalance, therefore, might disturb TRPM6 expression. Third, the basolateral membrane potential, maintained by the K^+^ permeability and Na^+^‐K^+^‐ATPase activity, provides the driving force for Na^+^ and Mg^2+^ extrusion. Depolarisation of the basolateral membrane potential in Na^+^ wasting disorders of the distal convoluted tubule may therefore lead to reduced activity of the putative Na^+^‐Mg^2+^ exchanger SLC41A1. Elucidating the interconnections between Mg^2+^ and Na^+^ transport in the distal convoluted tubule is hampered by the currently available models. Our analysis indicates that the coupling of Na^+^ and Mg^2+^ reabsorption may be multifactorial and that advanced experimental models are required to study the molecular mechanisms.

## 1 INTRODUCTION

The distal convoluted tubule (DCT) is an essential nephron segment for blood pressure regulation and potassium (K^+^) homeostasis. In the DCT, 10% of the filtered sodium (Na^+^) and magnesium (Mg^2+^) is reabsorbed in a transcellular mechanism,^1^ which is highly regulated by endocrine regulation.^2^, ^3^, ^4^, ^5^, ^6^, ^7^, ^8^, ^9^, ^10^ Genetic and acquired diseases of the DCT segment are therefore associated with renal Na^+^ and Mg^2+^ wasting. Notably, hereditary Na^+^ wasting disorders often present with hypomagnesaemia (serum Mg^2+^ <0.7 mmol L^−1^), a condition in which serum Mg^2+^ concentrations are below normal (normal 0.7‐1.05 mmol L^−1^). However, the mechanisms that explain hypomagnesaemia in these patients are largely unidentified. In this review, we present three hypotheses of mechanisms underlying the hypomagnesaemia caused by genetic DCT Na^+^ wasting disorders. In addition, we provide detailed descriptions on Na^+^ and Mg^2+^ reabsorption in the DCT.

### 1.1 Mechanisms of Na^+^ reabsorption in the DCT

**FIGURE 1:**
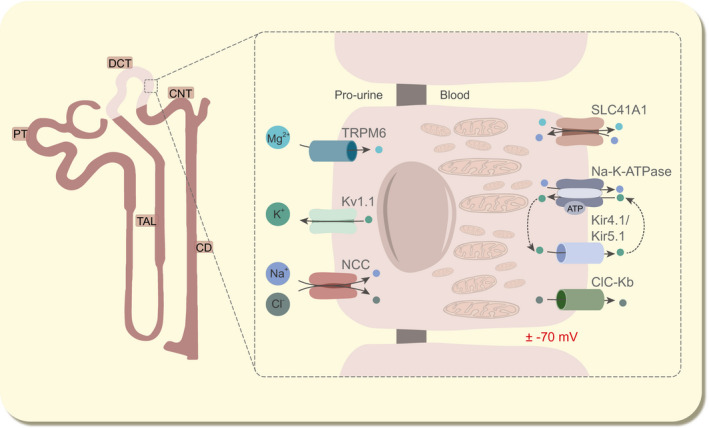
Electrolyte transport in the DCT. In a physiological condition, Mg^2+^ is reabsorbed into the cell by TRPM6 and is extruded into the blood compartment via SLC41A1 in exchange for Na^+^. Both Na^+^ and Cl^−^ are reabsorbed from the pro‐urine by NCC. At the basolateral side, Kir4.1/Kir5.1 channels are responsible for K^+^ extrusion, generating a negative membrane potential at ± 70 mV, which is maintained by the Na^+^‐K^+^‐ATPase using ATP. This K^+^‐recycling mechanism by the Kir4.1/Kir5.1 channels and Na^+^‐K^+^‐ATPase is called “pump‐leak coupling”. At the apical side, K^+^ is released to the pro‐urine by Kv1.1. Cl^−^ is extruded by ClC‐Kb to the blood. CD, collecting duct; ClC‐Kb, voltage‐gated Cl^−^ channel; CNT, connecting tubule; DCT, distal convoluted tubule; Kir4.1, K^+^ inwardly rectifying channel 4.1; Kv1.1, K^+^ voltage‐gated channel subfamily A member 1; NCC, Na^+^/Cl^−^ cotransporter; PT, proximal tubule; SLC41A1, solute carrier family 41 member 1, Na^+^‐Mg^2+^ exchanger; TAL, thick ascending limb; TRPM6, transient receptor potential melastatin 6

The DCT is responsible for the reabsorption of 5‐10% of the filtered Na^+^ load.^1^ Early micropuncture studies demonstrate that this may increase up to 30%‐45% when required, showing the enormous compensatory capacity of this segment.^11^ In the early DCT, apical Na^+^ uptake is facilitated by the thiazide‐sensitive sodium chloride (Cl^−^) co‐transporter (NCC) (Figure 1). Given that NCC is the sole Na^+^ transporter in the luminal plasma membrane in the DCT, decreased NCC activity results in renal Na^+^ wasting.^12^, ^13^ Basolateral Na^+^ extrusion towards the peritubular fluid depends on Na^+^‐K^+^‐ATPase activity. The activity of this pump and the high permeability of K^+^ via Kir4.1/Kir5.1 K^+^ channels set the basolateral membrane potential difference that typically ranges between −60 and −90 mV in the DCT.^14^, ^15^ Na^+^‐K^+^‐ATPase function depends directly on Mg^2+^‐bound ATP (Mg‐ATP) availability, and indirectly on free Mg^2+^ and the back‐leak of K^+^.^16^, ^17^, ^18^, ^19^, ^20^, ^21^ This so‐called “pump‐leak coupling” of K^+^ recycling between Na^+^‐K^+^‐ATPase is essential to maximise the Na^+^ reabsorption capacity of the DCT.^22^

Na^+^ reabsorption in the DCT depends on the number of NCC transporters present in the plasma membrane and subsequent activation by phosphorylation.^23^ Three residues in the intracellular N‐terminal region of NCC can be phosphorylated by Ste20‐like proline‐alanine–rich kinase (SPAK) and oxidative stress response kinase 1 (OSR1).^23^, ^24^ In turn, SPAK and OSR1 are activated by With‐No‐Lysine (WNK) kinases.^25^ WNK kinases are, therefore, the main target of pathways regulating Na^+^ reabsorption including, but not limited to, angiotensin II, vasopressin, insulin and aldosterone.^2^, ^3^, ^4^, ^5^, ^6^ Mutations in WNK1 and WNK4 are associated with familial hyperkaliaemic hypertension (FHHt) or pseudohypoaldosteronism type II (PHAII) (OMIM: 145260) as a result of increased NCC activity.^26^, ^27^, ^28^ Of note, most PHAII patients have mutations in ubiquitin ligase Cullin 3 (CUL3) or its adaptor protein Kelch‐like‐3 (KLHL3).^29^ The CUL3‐KLHL3 complex is essential for the ubiquitination of WNK1 and WNK4, thereby regulating their expression levels and indirectly determining NCC activity.^30^, ^31^

Recently, plasma K^+^ levels were identified as a major physiological determinant of NCC activity.^32^ A multitude of in vitro and in vivo studies have demonstrated that low extracellular K^+^ levels increase NCC phosphorylation independently of Na^+^ and angiotensin II levels.^33^, ^34^, ^35^, ^36^ These findings have resulted in the current model in which Kir4.1/Kir5.1 channels serve as a K^+^ sensor.^37^ K^+^ efflux via Kir4.1/Kir5.1 hyperpolarises the membrane and decreases the intracellular Cl^−^ concentration. Crystallography studies revealed that Cl^−^ binds to WNK kinases and thereby inhibits their autophosphorylation/activation.^38^, ^39^ Therefore, low [K^+^]_i_ ultimately results in the increase in NCC phosphorylation and thereby enhances Na^+^ reabsorption. This K^+^ sensing mechanism is the main determinant of the Na^+^ delivery to the aldosterone‐sensitive distal nephron, where the epithelial Na^+^ channel ENaC‐mediated Na^+^ uptake is coupled to K^+^ secretion via renal outer medullar K^+^ channel (ROMK). Na^+^ reabsorption in the DCT lowers the Na^+^ load in the CD, which allows retention of K^+^ via decreased ROMK‐mediated K^+^ secretion.^40^, ^41^, ^42^, ^43^, ^44^ As such, the DCT determines the downstream K^+^ handling and is an essential mediator of K^+^ homeostasis.

### 1.2 Mechanisms of Mg^2+^ reabsorption in the DCT

The DCT plays a crucial role in determining the urinary Mg^2+^ excretion as subsequent nephron segments cannot reabsorb Mg^2+^ from the pro‐urine. Transient receptor potential melastatin 6 (TRPM6) channels facilitate Mg^2+^ influx from the lumen (Figure 1).^45^ Each protein consists of 6 transmembrane domains and forms a tetramer to become functional at the apical membrane. Recent data suggest that TRPM6 requires heterotetramer formation with its family member TRPM7 to function.^46^, ^47^, ^48^, ^49^ The chemical gradient for Mg^2+^ is negligible (0.2‐1.0 mmol L^−1^ in the lumen vs 0.5‐1.0 mmol L^−1^ intracellular) and the TRPM6‐mediated Mg^2+^ influx is, therefore, dependent on the voltage gradient across the luminal membrane. It is postulated that this is orchestrated by the luminal voltage‐gated K^+^ channel Kv1.1.^50^, ^51^ The activity of TRPM6 is regulated by several external and internal factors, such as EGF, insulin, oestrogens, dietary Mg^2+^ intake and intracellular Mg^2+^ concentrations.^8^, ^9^, ^10^, ^52^, ^53^ Inactivating mutations in TRPM6 have been associated with hypomagnesaemia with secondary hypocalcaemia (HSH; OMIM: 602014).^54^, ^55^ In HSH patients, serum Mg^2+^ levels drop below 0.3 mmol L^−1^ and endanger proper brain development if left untreated.^54^

Unlike the influx of Mg^2+^ from the luminal side, the players facilitating Mg^2+^ efflux towards the blood compartment have not yet been conclusively elucidated. Two main mechanisms have been proposed, although they remain controversial. Mg^2+^ efflux towards the blood compartment requires an anti‐porter or ATPase, since no chemical gradient exists for Mg^2+^ while the voltage gradient favours Mg^2+^ influx. The presence of a Na^+^‐Mg^2+^ exchanger has been demonstrated as the mechanism for Mg^2+^ efflux in multiple cell types.^56^, ^57^ Although the molecular identity of the putative Na^+^‐Mg^2+^ exchanger has not been definitively identified, the most promising candidate is the solute carrier family 41 member 1 (SLC41A1). This transmembrane protein is located at the basolateral domain of the DCT and has been shown to facilitate Mg^2+^ efflux.^58^ Mutations in the gene have been observed in one patient of a consanguineous family suffering from a nephronophthisis‐like phenotype, although these patients do not experience hypomagnesaemia or renal wasting of magnesium.^59^ An alternative candidate for basolateral Mg^2+^ extrusion is cyclin M2 (CNNM2). This transmembrane protein localizes specifically to the basolateral compartment of the DCT and contains two cystathionine‐beta‐synthase (CBS) domains capable of binding Mg^2+^‐ATP.^60^, ^61^ Inactivating mutations have been implicated in a syndrome that prominently features hypomagnesaemia and renal magnesium wasting (OMIM: 613882).^14^, ^60^, ^62^ Although CNNM2 has been proposed as the Na^+^‐Mg^2+^ exchanger in the DCT, this hypothesis remains to be confirmed experimentally.^63^, ^64^, ^65^

## 2 Renal salt wasting disorders and hypomagnesaemia

Genetic disorders that reduce Na^+^ reabsorption in the DCT are associated with hypomagesaemia (Table 1). Patients with Gitelman syndrome, which is caused by mutations in the *SLC12A3* gene encoding the thiazide‐sensitive NCC (NCC; OMIM: 263800), suffer from renal Na^+^ wasting, hypokalaemia, metabolic alkalosis and hypomagnesaemia.^66^, ^67^, ^68^ A similar renal phenotype is observed in EAST/SeSAME syndrome caused by mutations in *KCNJ10* encoding Kir4.1 (OMIM: 612780).^16^, ^69^ Indeed, Kir4.1 determines NCC activity by indirectly affecting the [Cl^−^]_i_ and, in turn, WNK kinase activation.^37^, ^38^, ^39^ The hypokalaemia and metabolic alkalosis is likely caused by compensatory actions in the collecting duct (CD), where Na^+^ reabsorption is increased at the expense of K^+^ and H^+^ reabsorption. However, the mechanisms that explain hypomagnesaemia in these patients are largely unidentified.

**TABLE 1:** Overview of symptoms in DCT‐associated salt and Mg^2+^‐wasting syndromes. Including non‐DCT‐associated Bartter syndromes (type I, II and IV)

Syndrome	Classification	Gene	Protein	Type of mutation	Symptoms												References
					Aldosterone	Blood pH	Electrolytes										
							Blood					Urine					
							Ca^2+^	Cl^−^	K^+^	Mg^2+^	Na^+^	Ca^2+^	Cl^−^	K^+^	Mg^2+^	Na^+^	
Salt‐wasting disorders																	
TAL‐associated																	
Bartter syndrome	Type I	*SLC12A1*	NKCC2	LoF	↑	Alkalosis	=	=	↓	=	=	=	↑	=	↑	↑	71, 73, 74
	Type II	*KCNJ1*	ROMK	LoF	↑	Alkalosis	=	=	↓	=	=	=	↑	=	↑	↑	72, 73
	Type IV	*BSND*	Barttin	LoF	↑	Alkalosis	=	↓	↓	=/↓	=	↑	=	=	=	=	70, 73, 74
DCT‐associated																	
Bartter syndrome	Type III	*CLCNKB*	ClC‐Kb	LoF	=/↑	Alkalosis	=	↓	↓	↓	=	=	=	=	=	↑	73, 74, 75
EAST/SeSAME syndrome		*KCNJ10*	Kir4.1	LoF	↑	Alkalosis	=	=	↓	↓	=	↓	↑	↑	↑	↑	16, 69
Gitelman syndrome		*SLC12A3*	NCC	LoF	↑	Alkalosis	=	=	↓	↓	=	↓	=	=	=	↑	66, 67, 158, 159
Gordon syndrome or pseudohypoaldosteronism II		*WNK1* *WNK4*	WNK1 WNK4	GoF LoF	=	Acidosis	=	=/↑	↑	=	=	=/↑	=	=	=	=	28, 160, 161
		*CUL3*	CUL3	LoF	=	Acidosis	=	=/↑	↑	=	=	=/↑	=	=	=	=	29, 162
		*KLHL3*	KLHL3	LoF	=	Acidosis	=	=/↑	↑	=	=	=/↑	=	=	=	=	29, 162, 163
Mg^2+^‐wasting disorders^a^																	
Autosomal dominant hypomagnesaemia		*KCNA1*	Kv1.1	LoF	=	Normal	=	=	=	↓	=	=	=	=	↑	=	51, 164
Hypomagnesaemia with secondary hypocalcaemia syndrome		*TRPM6*	TRPM6	LoF	=	Normal	↓	=	=	↓	=	=	=	=	↑	=	54, 55, 165
Hypomagnesaemia, seizure and intellectual disability syndrome		*CNNM2*	CNNM2	LoF	=	Normal	=	=	=	↓	=	=	=	=	↑	=	60, 62
Isolated hypomagnesaemia		*FXYD2*	γ subunit of Na^+^‐K^+^‐ATPase	LoF	=	Normal	=	=	=/↓	↓	=	↓	=	=	↑	=	142, 143
		*ATP1A1*	α1 subunit of Na^+^‐K^+^‐ATPase	LoF	=	Normal	=	=	↓	↓	=	=	=	↑	↑	=	144

Abbreviations: ↑ indicates an increase; ↓ indicates a decrease; = indicates normality; ATP1A1, α1‐subunit of Na^+^‐K^+^‐ATPase; Barttin, Bartter syndrome, infantile, with sensorineural deafness; BSND, Barttin CLCNK type accessory beta subunit; CLCNKB & ClC‐Kb, voltage‐gated Cl^−^ channel; CNNM2, cyclin M2; CUL3, ubiquitin ligase Cullin 3; FXYD2, γ‐subunit of Na^+^‐K^+^‐ATPase; GoF, gain of function; KCNA1 & Kv1.1, K^+^ voltage‐gated channel family A member 1; KCNJ1, K^+^ voltage‐gated channel family J member 1; KCNJ10, K^+^ inwardly rectifying channel subunit J member 10; Kir4.1, K^+^ inwardly rectifying channel 4.1; KLHL3, kelch‐like‐3; LoF, loss of function; NCC, Na^+^/Cl^−^ cotransporter; NKCC2, Na‐K‐2Cl cotransporter; ROMK, renal outer medullary K^+^ channel; SLC12A1, solute carrier 12 member 1; SLC12A3, solute carrier 12 member 3; TRPM6, transient receptor potential melastatin 6; WNK1, with no lysine kinase 1; WNK4, with no lysine kinase 4.

^a^ A full overview of DCT associated Mg^2+^‐wasting disorder can be found in Viering et al (2017).^156^

Bartter syndrome is a hereditary disorder of Na^+^ reabsorption in the TAL, which is characterized by hypokalaemia, metabolic alkalosis, polyuria, hypercalciuria and nephrocalcinosis. Bartter syndrome is caused by mutations in *SLC12A1* encoding NKCC2 (type I), *KCNJ1* encoding ROMK (type II), *CLCNKB* encoding ClC‐Kb (type III) or *BSDN* encoding Barttin (type IV) (OMIM: 601678, 241200, 607364 and 602522, respectively).^70^, ^71^, ^72^, ^73^, ^74^, ^75^ Notably, hypomagnesaemia is not uniformly present in Bartter syndrome (Table 1). Hypomagnesaemia is generally only observed in Bartter syndrome type III and IV, in which patients can present with features of antenatal Bartter as well as Gitelman syndrome.^76^ Mice deficient for ClC‐Kb indeed shown hypermagnesuria, in line with the observed decreased serum Mg^2+^ concentrations in patients with type III Bartter. Generally, this phenomenon is explained by the expression pattern of ClC‐Kb and Barttin, which are not limited to TAL, but also present in the DCT. In line with this observation, the incidence of furosemide, an inhibitor of NKCC2, rarely results in hypomagnesaemia.^77^, ^78^, ^79^ Indeed, in an animal study, furosemide treatment did not result in hypomagnesaemia and was associated with increased TRPM6 expression in the DCT.^80^ Altogether we, therefore, hypothesise that the presence of hypomagnesaemia depends on reduced Na^+^ reabsorption in the DCT.

Congenital syndromes that impair Mg^2+^ reabsorption in the DCT, such as TRPM6 and CNNM2‐associated disorders, do not involve disturbances of Na^+^ or K^+^ homeostasis.^54^, ^55^, ^60^, ^62^ Drugs that reduce TRPM6 activity, eg EGFR inhibitors, cause hypomagnesaemia, but are not associated with increased Na^+^ wasting.^52^, ^81^, ^82^, ^83^, ^84^ Only drugs that affect both TRPM6 and NCC activity such as rapamycin and calcineurin inhibitors concomitantly result in Mg^2+^ and Na^+^ wasting.^85^, ^86^, ^87^ Altogether, these findings suggest that Na^+^ reabsorption affects Mg^2+^ reabsorption in the DCT but not *vice versa*. From a physiological point of view, this would mean that the Mg^2+^ reabsorption would be proportional to the Na^+^ reabsorption in the DCT. However, since Mg^2+^ homeostasis is also dependent on reabsorption in other nephron segments, bone storage and intestinal absorption, such correlations are rather complex to determine.

Given that patients with loss‐of‐function mutations in NCC or long‐term thiazide treatment suffer from hypomagnesaemia^66^, ^67^ and that both SPAK^−/−^ and NCC^−/−^ mice develop hypomagnesaemia,^13^, ^88^, ^89^, ^90^, ^91^ it is generally accepted that Mg^2+^ reabsorption is affected by Na^+^ reabsorption in the DCT. However, the nature of this relationship and the molecular mechanisms explaining this phenomenon are largely unknown. In the following part of this review, we will critically assess three mechanisms that may explain the link between Mg^2+^ reabsorption and NCC activity.

### 2.1 Does DCT remodelling affect Mg^2+^ reabsorption?

**FIGURE 2:**
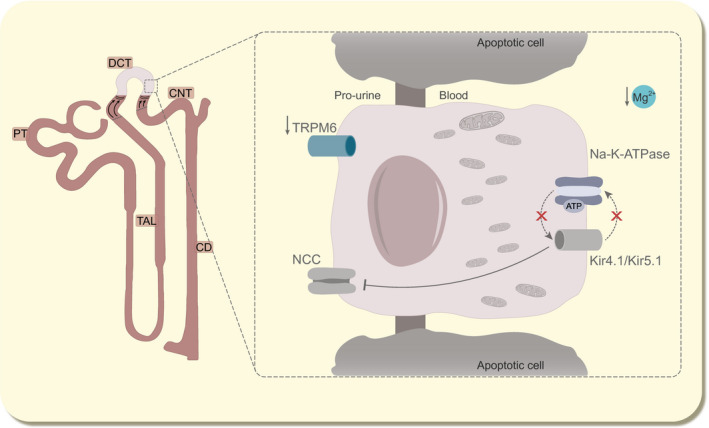
DCT remodelling affects the expression of TRPM6. Disturbed K^+^ recycling owing to the inactivating mutations in K^+^ channels (grey Kir4.1/Kir5.1) decreased Na^+^ reabsorption by NCC via the WNK/SPAK axis. Lowered NCC activity—inhibited or mutated (grey NCC)—leads to lowered K^+^ recycling and renal outer medullary K^+^ channel (ROMK)‐mediated K^+^ excretion in the CD. Consequently, due to the lowered Na^+^ reabsorption, the energy demand to fuel the Na^+^‐K^+^‐ATPase among others in form of ATP is reduced. This mechanism might cause a reduction in DCT cell mitochondrial mass and even apoptosis, which via an unknown mechanism leads to the shortening of the DCT segment. This will ultimately result in the overall decreased expression of TRPM6 and thereby lowered blood Mg^2+^ concentrations. OSR1, oxidative stress response kinase 1; P, phosphorylation; SPAK, Ste20‐like proline‐alanine rich kinase; WNKs, with no lysine kinases

NCC^−/−^ mice often serve as a model for Gitelman syndrome because they display similar features as patients, such as increased renin mRNA levels in kidney, hypomagnesaemia and hypocalciuria.^13^, ^91^ Since the first generation of NCC^−/−^ mouse, several groups have demonstrated atrophy of the DCT region,^12^, ^13^ suggesting that NCC activity is essential for DCT cell survival. Interestingly, TRPM6 expression is lowered in NCC^−/−^ mice and is accompanied by renal wasting of Mg^2+^,^92^ which potentially could be explained by structural differences in the DCT segment (Figure 2). Recently, Schnoz et al shown that NCC^−/−^ mice essentially lack DCT1 cells which has been attributed to an increase in apoptosis.^93^ Likewise, a mouse model suffering mutations found in Gitelman syndrome shown reduced early DCT mass.^94^ Consequently, a decrease in TRPM6 expression on protein level was observed. Yet, it cannot be excluded that the DCT cells, although less numerous, are capable to compensate by increasing TRPM6 activity at the cellular level.

Likewise, increased phosphorylation of NCC via gain‐of‐function (GoF) mutations in WNK4 in mice, which leads to PHAII in humans, has been shown to elongate the DCT and associated with a mild increase in serum Mg^2+^ levels.^95^ Similarly, mice with constitutively active SPAK (CA‐SPAK) display DCT hyperplasia and hypertrophy,^96^ while depletion of SPAK was associated with reduced DCT mass.^89^ This suggests that NCC activity, ie Na^+^ reabsorption in the DCT, is directly linked to DCT length. Interestingly, the GoF‐WNK4 mouse model shown impaired K^+^ secretion and hyperkalaemia which was attributed to increased NCC and reduced ENaC activity, resulting in diminished ROMK‐mediated K^+^ excretion.^95^ In contrast, it was reported that loss of Kir4.1, which leads to reduced NCC activity, is accompanied by a shortening of the DCT.^97^ In line, dietary K^+^ restriction resulted in increased phosphorylation of NCC as a result of increased Kir4.1 activity, and was accompanied by elongation of the DCT.^97^ Interestingly, long‐term use of furosemide, the inhibitor of NKCC2 in the TAL, has been associated with hyperplasia and hypertrophy in the DCT, CNT and CD.^11^, ^98^ Nevertheless, furosemide treatment generally does not result in hypermagnesaemia.^77^, ^79^, ^80^ However, it should be noted that furosemide decreases the driving force for Mg^2+^ reabsorption in the TAL, which may be compensated by increased Mg^2+^ reabsorption in the DCT. Moreover, increased renal Mg^2+^ reabsorption can be counteracted by reduced intestinal Mg^2+^ absorption or increased bone Mg^2+^ storage.

The mechanism by which altered Na^+^ or K^+^ load cues the DCT for adaptation remains obscure. It can, however, be hypothesized that DCT length is coupled to energy demand. The epithelial cells are packed with mitochondria owing to the need of ATP for proper Na^+^‐K^+^‐ATPase functioning. Lowered Na^+^ loads to the DCT will result in a decreased basolateral Na^+^ efflux and a decreased ATP requirement. Indeed, NCC^−/−^ DCT cells had decreased mitochondrial mass.^13^ In line, rats treated with thiazides demonstrated a decrease in cellular mitochondrial content, which was concomitant with a stimulation of apoptosis.^99^ Similarly, rats on enriched Na^+^ diets or on furosemide showed an increase in DCT volume and increase in mitochondrial content,^11^ associated with a higher metabolic demand of the cells.^100^, ^101^ Mitochondrial biogenesis, the process of producing more functional mitochondria, can be stimulated via pharmacological agents, such as AICAR or Rapamycin.^102^ It would be interesting to investigate if, under the right conditions, DCT shortening can be rescued via intervention of this mTOR‐AMPK pathway. It should be mentioned that Mg^2+^ reabsorption via TRPM6 has also been shown in vitro to be sensitive to mitochondrial activity. Electrophysiological analyses have shown that TRPM6 activity can be inhibited by H_2_O_2_, a by‐product of mitochondrial activity.^103^ Yet, other models are required to test its validity in vivo.

However, patients suffering hypertension and treated with thiazides already display an increased renal Mg^2+^ leakage within hours, suggestive that there are also acute responses at hand, eg hormonal, rather than DCT remodelling that modulate Mg^2+^ reabsorption in the DCT.^104^

### 2.2 Is Mg^2+^ reabsorption regulated via the same pathways that regulate the NCC?

The NCC phosphorylation cascade is well‐known for its sensitivity to hormones such as angiotensin II, aldosterone and insulin in order to maintain blood pressure.^2^, ^3^, ^4^, ^5^, ^6^ Interestingly, a number of paracrine and endocrine factors have been shown to regulate TRPM6.^7^, ^8^, ^9^, ^10^ Therefore, it can be speculated that there are common endocrine pathways that regulate both Na^+^ and Mg^2+^ reabsorption.

**FIGURE 3:**
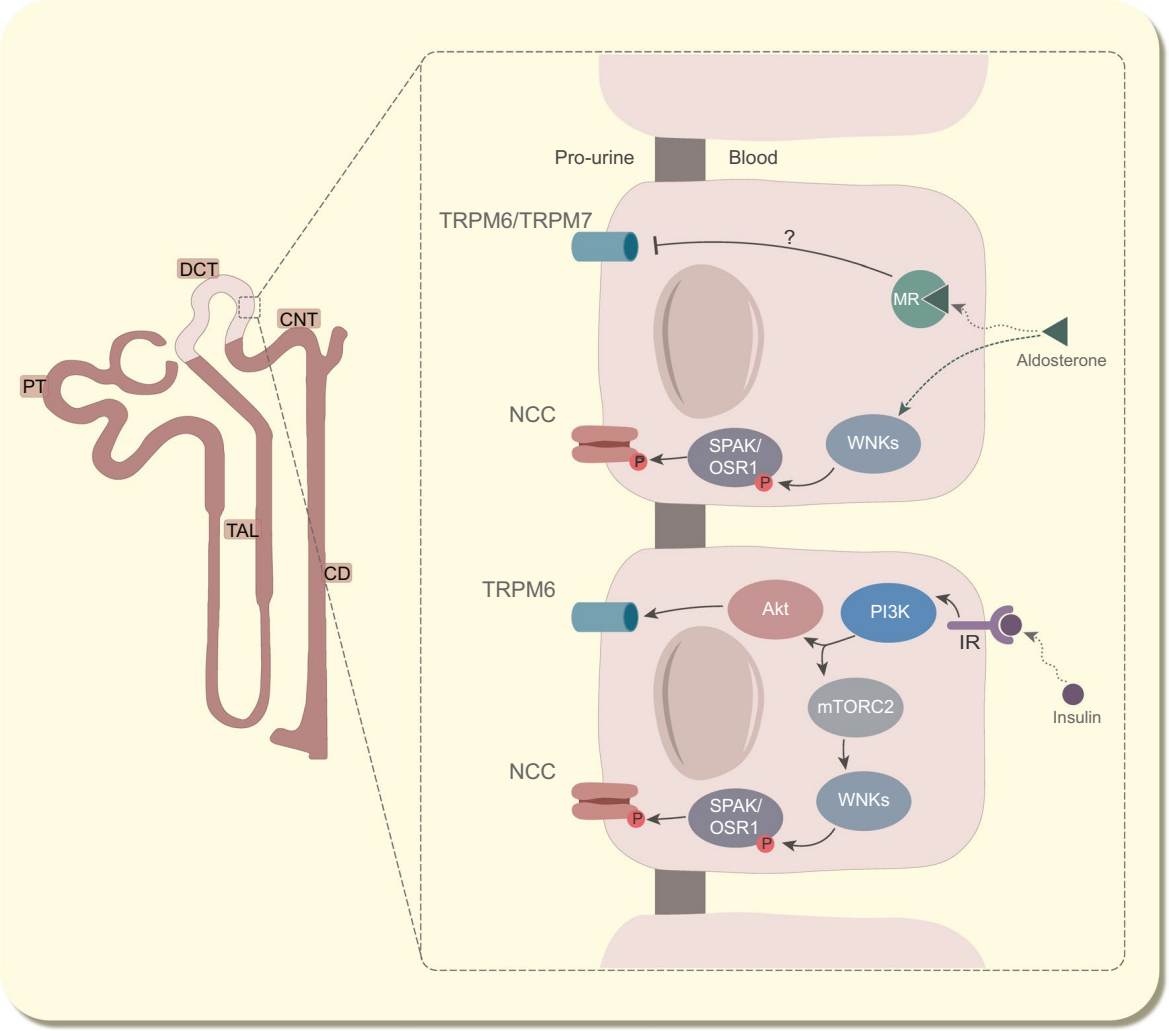
Dysregulation of NCC and TRPM6 common regulatory pathways. Inactivation of NCC frequently gives rise to secondary effects such as secondary hyperaldosteronism and insulin resistance in Gitelman patients. Under normal conditions, aldosterone (top) modulates NCC activation via MR/SGK1/NEDD4‐2 and WNK/SPAK axes. To regulate DCT Mg^2+^ reabsorption, aldosterone potentially acts on TRPM6/7 directly, although the mechanisms remain undetermined. Insulin (bottom) orchestrates NCC phosphorylation pathway by the PI3K/mTORC2 pathway while it modulates TRPM6 activity through the PI3K/Akt pathway. In Na^+^‐wasting disorders, hormonal disturbances will possibly dysregulate these signalling pathways, inhibiting TRPM6/7 activity in the process and ultimately resulting in hypomagnesaemia. Akt, protein kinase B; IR, insulin receptor; MR, mineralocorticoid receptor; mTORC2, mechanistic target of rapamycin complex 2; P, phosphorylation; PI3K, phosphoinositide 3‐kinases; SGK1, serum/glucocorticoid‐regulated kinase 1; TRPM7, transient receptor potential melastatin 7; Ub, ubiquitination

Aldosterone has been described as a regulator of both renal Na^+^ as well as Mg^2+^ reabsorption.^2^, ^3^, ^4^, ^105^, ^106^, ^107^ To regulate NCC, aldosterone targets the mineralocorticoid steroid receptor (MR) and stimulates SGK1 phosphorylation, which halts the E3 ubiquitin ligase NEDD4‐2, resulting in increased NCC activation .^108^ Moreover, it has been shown that aldosterone also increases the activity of WNK/SPAK axis indirectly by modulating blood K^+^ levels, although it is not fully understood how the two pathways interact (Figure 3).^2^, ^3^, ^4^, ^109^ Although the direct effect of this axis on the activity of TRPM6 has never been determined in vitro, van Megen et al have shown that DCT‐specific CA‐SPAK mice, in which NCC activity is increased, exhibit normomagnesaemia. Moreover, renal TRPM6 mRNA expression level was not altered.^110^ This suggests that TRPM6 regulation does not involve the WNK/SPAK axis and more direct pathways are likely involved.

Nevertheless, hypomagnesaemia and increased renal Mg^2+^ wasting have been described in patients suffering from hyperaldosteronism owing to the presence of primary adrenocarcinoma.^105^, ^111^ In rat models, aldosterone administration increased Mg^2+^ and Ca^2+^ levels in the urine and faeces, which was reversible upon spironolactone treatment, an antagonist of the aldosterone receptor.^112^, ^113^ It is, however, not clear whether changes in Mg^2+^ reabsorption are directly linked to decreased DCT‐mediated electrolyte reabsorption or if it is a systemic effect caused by changes in blood pressure.^112^ For instance, aldosterone administration in C57B6 mice was associated with decreased renal TRPM7 expression independent of changes in blood pressure, suggesting a direct effect of aldosterone on DCT Mg^2+^ reabsorption.^114^ On the other hand, hypertensive mice with an innate lowered serum Mg^2+^ levels displayed decreased TRPM6 expression upon aldosterone treatment, suggesting that these effects might be mediated by the changes in the extracellular volume.^115^ In addition to the difference in basal blood pressure levels, it is also important to note that the two mice models have different genetic backgrounds. Therefore, interpretation of results and conclusions drawing should be taken cautiously.

Currently, assessing the effect of aldosterone on TRPM6 function remains difficult because of the lack of cell models that express the protein endogenously. Nevertheless, the effect of aldosterone treatment on TRPM7 expression in the kidney has been studied in vitro. For instance, TRPM7 expression was increased via the SGK1‐mediated phosphorylation of the TRPM7‐kinase domain upon exposure of aldosterone in HEK293 cells and mediated Mg^2+^ influx, although these effects were not acute.^116^, ^117^ Yet, it is still not elucidated whether these results are specific for TRPM7 or could potentially be extended to TRPM6.^116^ Consequently, the effect of aldosterone on Mg^2+^ reabsorption in the kidney remains to be experimentally confirmed.

**FIGURE 4:**
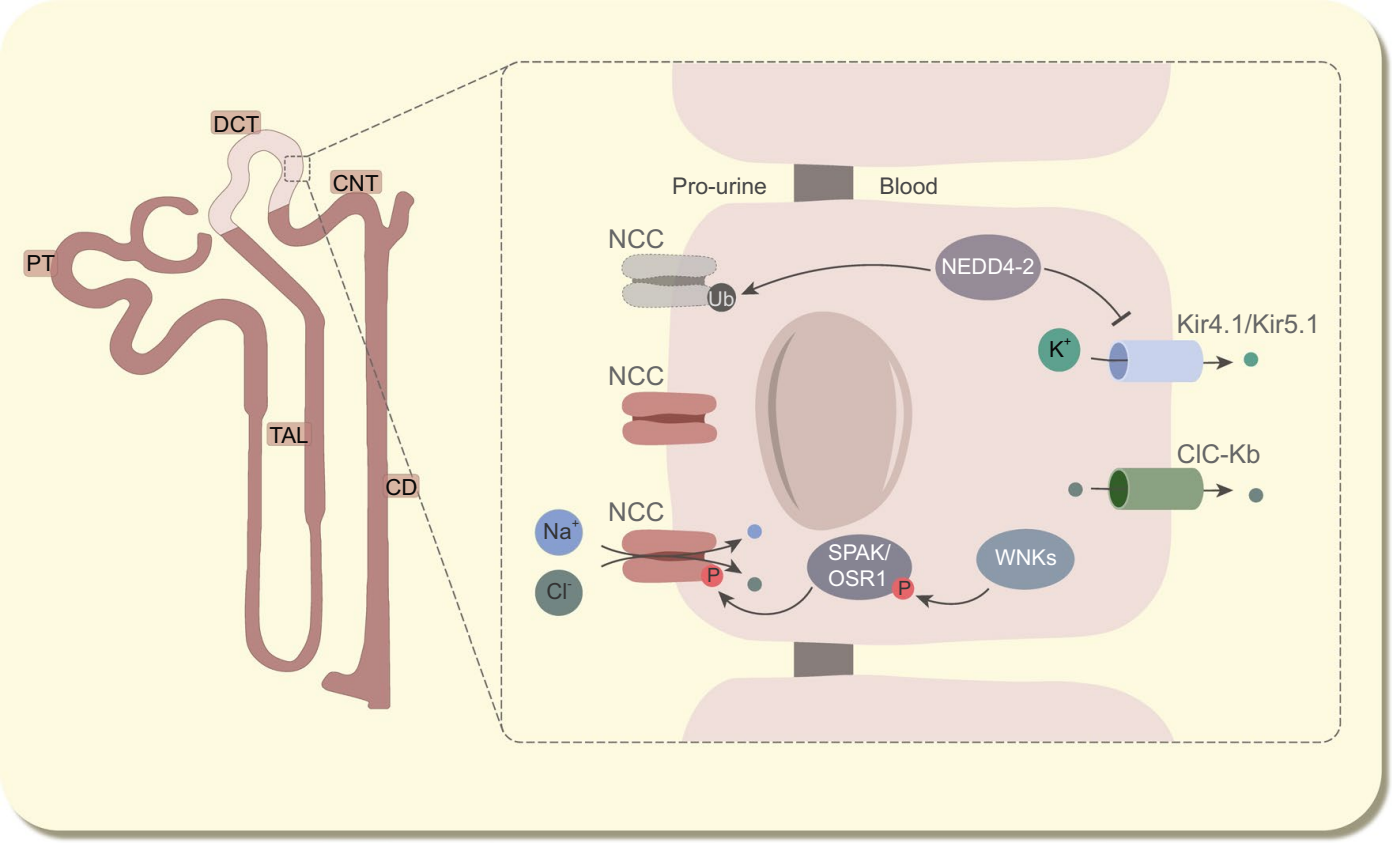
Model of NEDD4‐2 role in NCC activity. In normal condition, NCC activation and degradation is well‐orchestrated by the WNK/SPAK axis and the E3 ubiquitin ligase NEDD4‐2, respectively. Recently, NEDD4‐2 has also been shown to regulate basolateral K^+^ extrusion by ubiquitinating Kir4.1/Kir5.1. NEDD4‐2, neuronal precursor cell developmentally downregulated 4‐2

Interestingly, recent studies have disclosed that dietary depletion of Mg^2+^ can directly affect NCC‐mediated Na^+^ reabsorption. Ferdaus et al demonstrated that dietary Mg^2+^ restriction decreased the renal NCC expression.^118^ Unlike with K^+^ restriction diets, which leads to increased NCC phosphorylation via increased Kir4.1 activity,^119^ Mg^2+^ restriction led to degradation of NCC, possibly via the ubiquitin E3 ligase NEDD4‐2 (Figure 4). Mice deficient for NEDD4‐2 exhibited resistance to dietary Mg^2+^‐dependent NCC degradation. More recently, the same authors published a proposed mechanism by which NEDD4‐2 regulates Kir4.1/Kir5.1 function, which indirectly affects the intracellular Cl^−^ concentration, and thereby the WNK/SPAK‐axis.^120^ Whether the effects of Mg^2+^ were directed via Kir4.1/Kir5.1 was not explored. Free Mg^2+^ and Mg^2+^ bound ATP (Mg‐ATP) are known factors that inhibit TRPM6 function, as they can directly block channel activity.^121^, ^122^ How (intracellular) Mg^2+^ levels regulate NCC expression in the DCT should be experimentally investigated, since this could also aid in the understanding why patients suffering HSH or HSMR syndrome do *not* have altered Na^+^ reabsorption in the DCT.

Insulin stimulates Na^+^ reabsorption in the kidney, as notoriously known by the increased risk of hypertension in diabetic type II patients.^123^, ^124^ Apart from increasing Na^+^ transport in the proximal tubule and loop of Henle,^125^, ^126^ insulin has be shown to both modulate NCC and TRPM6 activity by a PI3K (phosphoinositide 3 kinases), mTORC2 (mechanistic target of rapamycin complex 2) and AKT1 (AKT serine/threonine kinase 1)‐dependent phosphorylation cascade (Figure 3).^5^, ^6^, ^10^ Although impaired glucose metabolism and insulin resistance have been described in Gitelman patients,^127^, ^128^, ^129^ the minor changes in plasma insulin levels make it unlikely that insulin is responsible for hypomagnesaemia in Na^+^ wasting disorders.

In addition to insulin and aldosterone, oestrogens has been shown to regulate TRPM6 and NCC expression.^130^, ^131^, ^132^, ^133^, ^134^, ^135^ For example, oestrogens increase renal NCC expression and activity via its phosphorylation^136^, ^137^ and TRPM6 mRNA levels in animal models.^53^, ^138^ Yet, no reports have been found that show a relationship between inactivating mutations in NCC and oestrogen level disturbances, making it unlikely that oestrogen affects DCT‐mediated Mg^2+^ reabsorption in patients with Na^+^ wasting disorders.

### 2.3 Could a depolarised membrane potential difference reduce Mg^2+^ reabsorption?

In the DCT, there is no chemical gradient for Mg^2+^ reabsorption since the extracellular and intracellular Mg^2+^ concentration are within the same range. TRPM6‐mediated Mg^2+^ influx in the DCT, therefore, depends solely on the electrical gradient.^51^ Consequently, maintaining the apical membrane potential difference is essential for Mg^2+^ reabsorption in this segment. Since Na^+^ and Cl^−^ co‐transport is electroneutral, and is not dependent on the apical membrane potential difference, it is unlikely that NCC directly affects TRPM6‐mediated Mg^2+^ transport. Studies in immortalized mouse DCT cells demonstrated that a reduced apical membrane potential significantly decreased Mg^2+^ uptake.^139^ It has been postulated that the apical K^+^ channel Kv1.1 contributes to the apical membrane potential difference, which would facilitate Mg^2+^ influx.^50^, ^51^, ^140^ Although direct membrane potential measurements in the DCT are technically challenging and therefore not available, a depolarised state of the apical membrane will inevitably result in a reduced driving force for apical Mg^2+^ transport via TRPM6.

**FIGURE 5:**
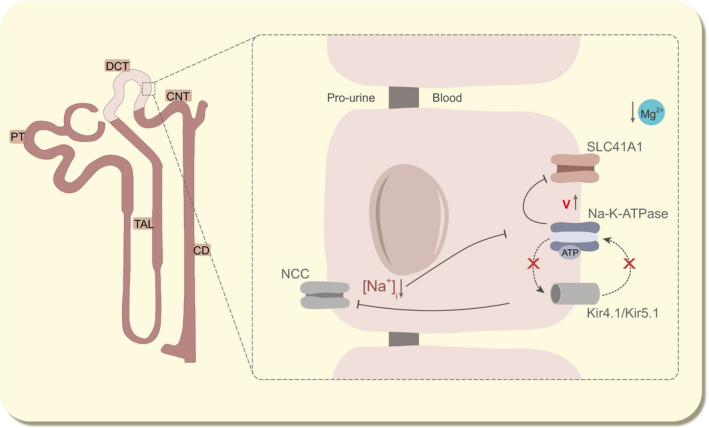
Loss of Na^+^‐K^+^‐ATPase activity hampers the activity of SLC41A1. NCC mutations (grey NCC) and Kir4.1 mutations (grey Kir4.1/Kir5.1) impede NCC activity causing decreased intracellular Na^+^ and reduced the Na^+^ supply to the Na^+^‐K^+^‐ATPase. In concert, “pump‐leak coupling” mechanism is uncoupled, abrogating Na^+^‐K^+^‐ATPase activity and thereby causing membrane depolarisation. Since basolateral Mg^2+^ extrusion at the basolateral side is proposed to be dependent on the Na^+^ gradient, this might ultimately impair Mg^2+^ efflux to the blood through the SLC41A1. [Na^+^]_i_, intracellular Na^+^ concentrations. V, membrane potential

The Na^+^‐K^+^‐ATPase plays a central role in DCT physiology, specifically in electrogenic ion transport (Figure 1). The DCT has the highest activity of this heterodimer within the kidney, which is accompanied with the highest density of mitochondria as generator of ATP.^141^ The Na^+^‐K^+^‐ATPase provides the driving force that is required for NCC activity, and sets the basolateral membrane potential difference at ±−70 mV. Mutations in *ATP1A1* and *FXYD2*, encoding the alpha and gamma subunits, respectively, of the Na^+^‐K^+^‐ATPase have been associated with hypomagnesaemia and renal Mg^2+^ wasting.^142^, ^143^, ^144^ Moreover, prolonged treatment with Na^+^‐K^+^‐ATPase inhibitors increased the incidence of hypomagnesaemia.^145^, ^146^ These findings highlight the importance of the Na^+^‐K^+^‐ATPase for renal Mg^2+^ reabsorption (Figure 5).

Salt‐wasting disorders of the DCT indirectly cause decreased Na^+^‐K^+^‐ATPase activity. As Kir4.1 is essential for basolateral K^+^ recycling at the basolateral membrane, Kir4.1 mutations that cause EAST/SeSAME syndrome, impair Na^+^‐K^+^‐ATPase activity.^16^, ^69^, ^147^ By uncoupling the “pump‐leak mechanism” at the basolateral membrane, the plasma membrane will be depolarised via reduced Kir4.1 K^+^ extrusion. This would limit the Cl^−^ extrusion via ClC‐Kb, lead to an increased intracellular Cl^−^ concentration, the inhibition of WNK kinases, and ultimately inhibited NCC‐mediated Na^+^ reabsorption. Indirectly, changes in the basolateral membrane potential could thereby regulate NCC function. On the other hand, interestingly, although Na^+^‐K^+^‐ATPase activity has never been directly assessed in Gitelman syndrome, data from thiazide‐treated rats demonstrate reduced Na^+^‐K^+^‐ATPase activity in the DCT.^148^ Upon thiazide treatment, the reduced NCC activity may decrease the intracellular Na^+^ in the DCT, reducing the Na^+^ supply to the Na^+^‐K^+^‐ATPase. Indeed, Na^+^‐K^+^‐ATPase activity in the proximal tubule and loop of Henle was not altered by thiazide treatment.^148^

Given that the Na^+^‐K^+^‐ATPase is crucial for the K^+^ recycling and thereby contributes to K^+^ permeability, its reduced activity in EAST/SeSAME and Gitelman syndrome will result in a depolarised basolateral membrane. Basolateral Mg^2+^ extrusion is generally considered to be Na^+^ dependent. A wide range of experiments in different cell types have demonstrated the presence of a Na^+^‐Mg^2+^ exchange mechanism.^149^ Reduced Na^+^‐K^+^‐ATPase in salt‐wasting syndrome of the DCT may, therefore, directly reduce the Na^+^ gradient that is required for Mg^2+^ extrusion. Although the exact molecular identity of the Mg^2+^ extrusion mechanism is under debate, Kolisek and colleagues have advocated that SLC41A1 functions as Na^+^‐Mg^2+^ exchanger in a 2:1 stoichiometry.^150^, ^151^ However, the Na^+^ dependence of Mg^2+^ efflux via SLC41A1 is under debate.^150^ Arjona et al recently showed that SLC41A1 facilitates Na^+^ and Cl^−^ independent Mg^2+^ efflux in overexpression models.^152^ Further studies in native DCT cells are required to further elucidate this mechanism. The nature of the Mg^2+^ extrusion mechanism is important to understand the effects of Gitelman and EAST/SeSAME syndrome on Mg^2+^ reabsorption.

## 3 Conclusion and perspectives

Na^+^ and Mg^2+^ reabsorption in the DCT are closely coupled. Atrophy of the DCT caused by loss of NCC activity is the most supported hypothesis to explain hypomagnesaemia in Na^+^ wasting disorders. Although these data are mainly obtained in animal models and biopsies of Gitelman patients are rarely executed, recent data suggest that progressive regression of the DCT explains the late clinical onset of the syndrome.^93^ However, hormonal pathways that co‐regulate NCC and TRPM6 and the effects of changed basolateral Na^+^ and K^+^ transport cannot be excluded and may also contribute to hypomagnesaemia.

In conclusion, further studies should provide final answers on the coupling of Na^+^ and Mg^2+^ reabsorption of the DCT. Our comprehensive analysis shows that this process is not dependent on a single factor, emphasizing the complexity of experimental design mimicking physiologically representative conditions. Recent advances in kidney organoid cultures may provide an advanced tool to dissect how Mg^2+^ transport is dependent on NaCl reabsorption, as they provided insights in other congenital disorders.^153^, ^154^, ^155^ Dissecting the underlying molecular mechanisms would not only add to the fundamental knowledge of ion transport in the kidney but it would also be an invaluable addition towards understanding the development of hypomagnesaemia in inherited Na^+^ wasting disorders.

## CONFLICTS OF INTEREST

None.
